# Transient elevation of serum ferritin in a Sri Lankan with homozygosity for H63D mutation in the *HFE* gene: a case report

**DOI:** 10.1186/s13256-020-02428-3

**Published:** 2020-07-09

**Authors:** Wasanthi Wickramasinghe, Chathurika Karunathilaka, Saroj Jayasinghe, Lallindra Gooneratne

**Affiliations:** 1 Clinical Haematology, Faculty of Medicine, University of Colombo, Colombo, Sri Lanka; 2 Clinical Haematology, National Hospital of Sri Lanka, Colombo, Sri Lanka; 3 Department of Pathology, Faculty of Medicine, University of Colombo, Colombo, Sri Lanka; 4 Haematology, Faculty of Medicine, University of Colombo, Colombo, Sri Lanka

**Keywords:** Hereditary hemochromatosis, H63D homozygosity, Sri Lanka, Venesection, Case report

## Abstract

**Introduction:**

Hereditary hemochromatosis is an inherited disorder of iron metabolism, characterized by excessive iron deposition in major organs of the body, leading to multi-organ dysfunction. It is a genetically heterogeneous disease caused by mutations in one or more different genes, the most common being mutations in the *HFE* gene. *HFE* hereditary hemochromatosis is mostly found in Europeans and is almost always a result of two mutations: C282Y and H63D. The H63D mutation is not as penetrant as the C282Y mutation, but there are rare reported cases of hereditary hemochromatosis with homozygous H63D genotype. While the C282Y mutation is primarily confined to persons of Northern European origin, the H63D mutation is spread worldwide. Other types of hereditary hemochromatosis are rare and broadly defined as non-*HFE* hereditary hemochromatosis and include mutations in the hemojuvelin gene, hepcidin (*HAMP* gene), transferrin receptor 2 gene, and ferroportin gene.

Hereditary hemochromatosis is commonly found in populations of European origin; in contrast, it is rare and less well understood in Asia. It can be masked by the presence of concurrent iron deficiency or secondary iron overload in thalassemias.

**Case presentation:**

We report the case of a 42-year-old Sri Lankan man investigated for fatigue during a brief upper respiratory tract infection and found to have high liver transaminases and high serum ferritin, which persisted even after complete resolution of the infection. Homozygosity for H63D mutation in the *HFE* gene was detected. Liver enzymes, serum ferritin, and transferrin saturation normalized following venesections.

**Conclusion:**

This case adds to the literature on the importance of being vigilant and investigating patients suspected for iron overload, including genetic studies for hereditary hemochromatosis, even though it is a rare clinical entity in Asians.

## Introduction

Hereditary hemochromatosis (HH) is an inherited disorder of iron metabolism characterized by excessive iron deposition in major organs of the body, leading to multi-organ dysfunction [1]. It is a genetically heterogeneous disease caused by mutations in one or more different genes, the most common being mutations in the *HFE* gene (type 1 HH) [2]. *HFE* HH is mostly found in Europeans and is almost always a result of two mutations: C282Y and H63D [3]. A third mutation was identified at amino acid 65 (S65C), however, it is less common and infrequently associated with iron overload [4]. The H63D mutation is not as penetrant as the C282Y mutation, but there are rare reported cases of HH with homozygous H63D genotype [5]. H63D mutations may lead to iron overload in the setting of another risk factor, such as beta-thalassemia trait [6] or hepatitis C [7]. While the C282Y mutation is primarily confined to persons of Northern European origin, the H63D mutation is spread worldwide [8]. Other types of HH are rare and broadly defined as non-*HFE* HH, and include mutations in the hemojuvelin gene, hepcidin (*HAMP* gene), transferrin receptor 2 gene, and ferroportin gene [5].

HH is commonly found in populations of European origin and, in contrast, is rare and less well understood in Asia. It can be masked by the presence of concurrent iron deficiency or secondary iron overload in thalassemias [2].

We report the case of a 42-year-old Sri Lankan man investigated for fatigue during a brief upper respiratory tract infection, and found to have high liver transaminases and high serum ferritin that persisted even after complete resolution of the infection. Homozygosity for H63D mutation in the *HFE* gene was detected. Liver enzymes, serum ferritin, and transferrin saturation normalized following venesections.

This case adds to the literature on the importance of being vigilant and investigating patients suspected for iron overload, including genetic studies for HH, even though it is a rare clinical entity in Asians.

## Case report

A 42-year-old, previously healthy Sri Lankan man presented with low-grade fever, upper respiratory symptoms, and fatigue of 1-week duration. Investigations revealed elevated liver enzymes: aspartate aminotransferase (AST) 117 U/L and alanine aminotransferase (ALT) 186 U/L. There was no history of jaundice, pruritus, or alcohol use. An ultrasound scan of his abdomen showed grade 1 fatty liver with no liver parenchymal changes. Viral screening for hepatitis A, B, and C were negative. Serum ferritin was 1292 μg/L. Full blood count was normal, including hemoglobin (Hb) 147 g/L, white blood cells (WBC) 6.5 × 10^9^/L, and platelets 213 × 10^9^/L; C-reactive protein (CRP) was 4.8 mg/dL, total bilirubin was 15.9 μmol/L, total protein was 74 g/L, and albumin was 44 g/L, which were all normal. Serum ferritin repeated in a month, after complete resolution of the illness, was 1103 ng/mL, with a transferrin saturation of 75%, and liver enzymes remained moderately elevated (AST 83.4 U/L, ALT 155 U/L). There was no consanguinity in his parents and no family history of HH. Investigation in regards to inherited iron loading conditions were considered after excluding secondary causes of iron overload. Genetic testing for p.H63D and p.C282Y mutations in the *HFE* gene by DNA extraction, allele-specific polymerase chain reaction (PCR), and agarose gel electrophoresis showed that he was homozygous for the H63D mutation. A liver biopsy showed increased hepatocellular iron content and features of steatohepatitis. Investigations to assess complications of iron overload, including fasting blood sugar, electrocardiography, and two-dimensional echocardiography, were normal. He was started on venesections. Following two venesections done 2 weeks apart, his liver enzymes started to decline slowly (AST 76 U/L, ALT 151 U/L), serum ferritin came down to 200 μg/L, and transferrin saturation to 26%.

## Discussion

*HFE* hemochromatosis is an autosomal recessive disease, the most common cause of hemochromatosis in Northern Europe with a prevalence of 1 in 200–400 people of European ancestry [9]. The *HFE* gene encodes for a novel 343 amino acid major histocompatibility complex (MHC) class 1 molecule. Initially, two missense mutations in the *HFE* gene, located on chromosome 6q, were identified, namely C282Y and H63D [3]. H63D mutation, except in very rare instances, does not occur on the same allele as C282Y [10]. A third mutation resulting in a serine to cysteine substitution at amino acid 65 (S65C) has also been described more recently, which is said to result in a mild form of HH [11]. There are at least another 38 allelic variants of the *HFE* gene [12–14]; however, most do not appear to be clinically significant.

HH is uncommon in Asian populations and the C282Y mutation is rarely found [2], whereas the H63D mutation which is spread worldwide is seen more frequently.

Transferrin saturation and serum ferritin are significantly higher in individuals with C282Y homozygous hemochromatosis than other individuals [15]. Homozygosity for H63D is usually not associated with the development of clinically significant iron overload, but homozygosity and heterozygosity may be associated with a significant increase in serum ferritin and transferrin saturation compared to wild type H63D [16].

A study done in Canada by Samarasena *et al.* showed H63D homozygotes have elevated transferrin saturation compared to the wild genotype [17]. This was comparable to C282Y homozygotes and compound heterozygotes; however, the clinical significance of this finding is unclear and warrants further studies [17].

The overall clinical significance of H63D mutation remains unclear, and some postulate that it plays a role in the development of the disease, but the penetrance tends to be low [18–23]. Some studies have suggested that H63D mutation is simply a polymorphism and may not be of clinical significance when present without any other mutations [17, 24, 25]. A retrospective analytical study done in Canada from 1999 to 2009 suggested a low penetrance of H63D homozygosity. Furthermore, it showed that although serum ferritin levels were elevated at baseline, there was no significant increase in mean ferritin level in the cohort over time [26]. Only three individuals met the criteria for iron overload at the time of genotyping and four at follow up. Within this group, only one individual met the criteria for iron overload-related disease, and he was documented to have alcohol abuse, so that it is not clear whether iron overload was the primary cause of liver disease. Neghina and Anghel [27] conducted a meta-analysis examining the association between the various *HFE* genotypes and iron overload and found that in individuals with a clinical diagnosis of HH, the C282Y homozygous genotype had a 100 times greater association with an elevated transferrin saturation, serum iron, and serum ferritin when compared with H63D homozygotes. However, some studies have shown the H63D mutation to be associated with clinically significant iron overload. One study [26] found that 3 out of 61 individuals with HH phenotype were homozygous for H63D mutation, however, not all of these patients underwent liver biopsies and, in some, the phenotypic diagnosis was based solely on the elevated transferrin saturation.

Some have postulated that individuals with H63D homozygotes who develop clinically significant iron overload may have another unknown contributing factor, either a mutation or an environmental factor [26]. Aranda *et al.* [28] found that iron intake, alcohol intake, tobacco, and male sex were all positively associated with elevated iron studies in all *HFE* genotypes, including C282Y, H63D, and S65C homozygotes.

Our patient’s liver biopsy showed mildly increased hepatocellular iron content and features of steatohepatitis. In a large multicenter US cohort of patients with the hepatic phenotype of HH, hepatic iron concentration was significantly higher among C282Y homozygotes than in C282Y/ H63D compound heterozygotes. Also, the patients who were not homozygous for C282Y were more likely to have Kupffer cell iron, hepatic necroinflammation, and steatosis [4]. This suggests that expression of the hepatic HH genotype among non-C282Y homozygotes is associated with the presence of a “second hit” that contributes to liver disease leading to hepatic iron overload. In a study done in the UK, of 366 blood samples referred for genetic analysis of the *HFE* gene, four patients were homozygous for H63D mutation. All four showed fatty liver on an ultrasound scan, while three had mildly elevated liver enzymes [29].

In a study done on the frequencies of the *HFE* genotypes in the general populations of many countries, the incidence of C282Y homozygosity and heterozygosity was 0% in Sri Lanka, while the incidences of H63D homozygosity and heterozygosity were 0.9% and 16.5% respectively [5]. A Sri Lankan study on *HFE* gene analysis in 125 patients with thalassemia major in 2012 showed an allele frequency of 0% for C282Y and of 9.2% for H63D [30]. However, there was no statistically significant association with serum ferritin level between the heterozygous group and the wild type allele of H63D mutation. In another study done with Sri Lankan families, H63D was found on three new haplotypes and C282Y on one new haplotype, and it demonstrated a new C282Y mutation, suggesting that both mutations in Sri Lanka had arisen independently from the mutations occurring in Europe [31].

Our patient presented with vague nonspecific symptoms and was found to have raised liver enzymes, high serum ferritin, and high transferrin saturation, and a liver biopsy showed increased hepatocellular iron content and features of steatohepatitis, and genetic studies showed homozygosity for H63D mutation. His very high serum ferritin level could be due to variable presentations of homozygous H63D mutation itself, or the presence of other mutations contributing to the phenotype, or transient environmental risk factors such as viral hepatitis. After just two venesections his serum ferritin level came down to 200 μg/L and over a month his liver enzymes also came down; we can hypothesize that his initial presentation is most likely due to an associated additional risk factor in the background of homozygous H63D mutation. We have planned regular clinic follow up for our patient with monitoring of liver enzymes, serum ferritin, and transferrin saturation. Furthermore, family screening is necessary to identify other affected family members.

## Conclusion

Because of its protean manifestations, hemochromatosis should be considered in any patient of European descent presenting with fatigue, arthritis, liver dysfunction, gonadal failure, diabetes mellitus, and skin pigmentation. However, in the relevant clinical background, it should be suspected and evaluated in the South Asian population as well.

## Data Availability

Not available.
